# Characteristics of Clinically Classified Oral Lichen Planus in Optical Coherence Tomography: A Descriptive Case-Series Study

**DOI:** 10.3390/diagnostics13162642

**Published:** 2023-08-10

**Authors:** Yuliia Gruda, Marius Albrecht, Michaela Buckova, Dominik Haim, Guenter Lauer, Edmund Koch, Korinna Joehrens, Christian Schnabel, Jonas Golde, Jiawen Li, Robert A. McLaughlin, Julia Walther

**Affiliations:** 1 Carl Gustav Carus Faculty of Medicine, Department of Medical Physics and Biomedical Engineering, TU Dresden, Fetscherstraße 74, 01307 Dresden, Germany; yuliia.gruda@mailbox.tu-dresden.de (Y.G.); marius.albrecht@mailbox.tu-dresden.de (M.A.); christian.schnabel@tu-dresden.de (C.S.); jonas.golde@tu-dresden.de (J.G.); 2 Carl Gustav Carus Faculty of Medicine, Institute of Pathology, TU Dresden, Fetscherstraße 74, 01307 Dresden, Germany; korinna.joehrens@uniklinikum-dresden.de; 3 Carl Gustav Carus Faculty of Medicine, Clinic and Policlinic of Oral- and Maxillofacial Surgery, TU Dresden, Fetscherstraße 74, 01307 Dresden, Germany; michaela.buckova@uniklinikum-dresden.de (M.B.); dominik.haim@uniklinikum-dresden.de (D.H.); guenter.lauer@uniklinikum-dresden.de (G.L.); 4 Carl Gustav Carus Faculty of Medicine, Department of Anesthesiology and Intensive Care Medicine, Clinical Sensoring and Monitoring, TU Dresden, Fetscherstraße 74, 01307 Dresden, Germany; edmund.koch@tu-dresden.de; 5 Faculty of Sciences, Engineering and Technology, The University of Adelaide, Adelaide 5005, Australia; jiawen.li01@adelaide.edu.au; 6 Institute for Photonics and Advanced Sensing, The University of Adelaide, Adelaide 5005, Australia; robert.mclaughlin@adelaide.edu.au; 7 Faculty of Health and Medical Sciences, The University of Adelaide, Adelaide 5005, Australia

**Keywords:** optical coherence tomography, biomedical imaging, fiber optics, endoscopy, human oral mucosa, oral lichen planus, leukoplakia, histology, precancerous lesions, oral cancer

## Abstract

Malignant transformation of oral lichen planus (OLP) into oral squamous cell carcinoma is considered as one of the most serious complications of OLP. For the early detection of oral cancer in OLP follow-up, accurate localization of the OLP center is still difficult but often required for confirmatory biopsy with histopathological examination. Optical coherence tomography (OCT) offers the potential for more reliable biopsy sampling in the oral cavity as it is capable of non-invasively imaging the degenerated oral layer structure. In this case-series study with 15 patients, features of clinically classified forms of OLP in OCT cross-sections were registered and correlated with available histologic sections. Besides patients with reticular, atrophic, erosive and plaque-like OLP, two patients with leukoplakia were included for differentiation. The results show that OCT yields information about the epithelial surface, thickness and reflectivity, as well as the identifiability of the basement membrane and the vessel network, which could be used to complement the visual clinical appearance of OLP variants and allow a more accurate localization of the OLP center. This forms the basis for further studies on OCT-assisted non-invasive clinical classification of OLP, with the aim of enabling decision support for biopsy sampling in the future.

## 1. Introduction

Any permanent oral lesion can lead to oral cavity cancer [1]. Therefore, early diagnosis and continuous surveillance, especially of suspicious lesions, are crucial [2,3]. It has to be taken into account that the prevalence of oral lesions and malignancies increases with age [4]. Thus, as a result of demographic change, the prevalence of oral cavity cancer is expected to rise further from the current level of approximately 380,000 cases per year worldwide [2,3]. One of the most common oral mucosal diseases leading to lesions and invasive cancer is oral lichen planus (OLP), with a prevalence up to 2% in dental practice [5,6,7]. Based on chronic inflammation, this disease can also affect the skin, the scalp and the genital areas [8,9]. However, the oral form of lichen planus is primarily relevant as it leads to malignancy in about 1–2% of cases, which is more frequent than the cutaneous form [10,11,12,13,14,15]. This applies in particular if additional risk factors such as smoking, alcohol consumption or an infection with HPV are present [16]. In general, the risk of progression tends to be higher for the red form of OLP [12,13,14]. Nevertheless, a long-term cohort study from Arduino et al. [12] also reported invasive oral cancer arising from the white form.

With regard to the current clinical management of OLP, the initial diagnosis is predominantly based on visual findings of the characteristic clinical features of OLP, which can be confirmed by oral biopsy examination with histopathology in conspicuous cases to exclude malignancy [17,18]. For the regular follow-up of OLP patients, biopsies are recommended as soon as progressive development with suspicious features is visually evident. Since clinically manifested OLP can change over time and mimic leukoplakia, the differential diagnosis of both entities is often difficult to discriminate visually as well as histopathologically [17]. If malignancy is suspected in the OLP follow-up examination due to high-risk progressive dysplastic and/or symptomatic alteration, the current gold standard for the diagnosis of oral cancer and its pre-stages includes anamnesis and clinical examination complemented by tissue biopsy and histopathological evaluation [19,20]. The form of OLP is not a definitive factor for or against the decision to take a biopsy. Moreover, the selection of suitable regions for the biopsy, especially in cases of multiple or widespread lesions, as well as the constant dynamic development of lesions, is challenging [19,21]. For this reason, intensive research into alternatives to the diagnostic approach has been conducted for several years. Considering that conventional methods such as sonography or magnetic resonance imaging (MRI) do not possess the necessary resolution to adequately visualize the architecture of the oral mucosa [22], new non-invasive optical imaging techniques are of great interest. Among these, optical coherence tomography (OCT) is particular noteworthy because, unlike other methods like autofluorescence or narrow band imaging [23,24], it enables real-time high-resolution cross-sectional imaging in depth [25,26] and may serve as a preinvasive diagnostic tool that supports tissue biopsy with increased accuracy [21,27]. A notable advantage of OCT lies in the possibility of visualizing the layered structure of normal oral soft tissue in vivo [28,29], as well as its degeneration in the case of pathological changes associated with oral cancer [30,31,32,33]. In fact, previous studies demonstrated that OCT can detect oral cancer with a sensitivity of approximately 92% and differentiate it from healthy mucosa with a specificity of approximately 94% [34,35]. Furthermore, with regard to epithelial dysplasia, a moderate specificity of 76% was reported [36]. Here, OCT was not able to distinguish between the different stages of dysplasia with certainty [36]. Concentrating on the OCT imaging of oral lesions in general, it has to be mentioned that lesions caused by OLP have only been studied occasionally so far [37,38,39]. Furthermore, with regard to OCT images, there is no systematic description of the key features of the different forms of OLP.

In this case-series study, we present the results of the depth-resolved OCT imaging of visually diagnosed OLP of the human oral mucosa in vivo during follow-up examination by means of the clinical use of a customized fiber-based probe [40]. For differentiation, we also included two patients with visually diagnosed leukoplakia with and without dysplastic change in our study. Based on the clinical classification of OLP, OCT features of different OLP types and subtypes were identified and correlated with the clinical appearance for all patients as well as histological sections in two clinical cases. To the best of our knowledge, for the first time, we demonstrate detailed descriptive in vivo OCT features for clinically classified forms of OLP distinct from leukoplakia based on a small number of patients.

## 2. Materials and Methods

In order to perform accurate follow-up diagnosis of patients with OLP or other pathological changes leading to oral cancer, a miniaturized OCT probe was tested in a clinical study. The study was authorized by the ethics commission of the Faculty of Medicine of the TU Dresden (EK 96032018 2018-11-16 and EK 39112018 2019-01-22).

### 2.1. OCT Miniprobe

**Figure 1:**
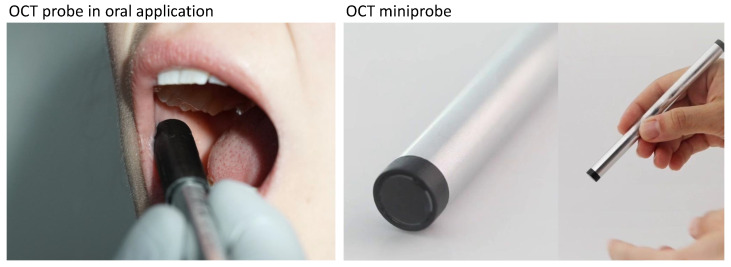
Application of the OCT probe in the human oral cavity in vivo by means of OCT imaging of the buccal mucosa of a healthy subject for demonstration. The OCT miniprobe is covered with a dental disposable plastic cover (X-ray Cover, Henry Schein Inc., Melville, NY, USA) fixed by an one-use cover sleeve (see **left**). Customized OCT miniprobe in the overall and detailed view of the distal end of the probe without disposable dental cover for single use in the oral cavity (see **right**).

For the imaging of OLP and leukoplakia of the human oral mucosa, we used the fiber probe OCT system described in [40] clinically on 13 patients with OLP and 2 patients with leukoplakia for comparison. The scanning optics of the miniprobe consists of a customized spliced GRIN fiber optic that is laterally deflected by a magnetic scanning system based on a ring magnet (1 mm outer diameter; attached to the fiber optic) and three copper wire coils which are used to drive the scanning motion of the fiber probe. The fiber probe with a scanning system is located inside a stainless-steel cylinder (10 mm diameter, 140 mm length) with a 4${}^{\circ }$ angled sapphire glass window (8 mm × 0.15 mm (diameter × width), UQG Optics Ltd., Cambridge, UK) at the proximal end of the probe (right part in Figure 1) [40]. The miniprobe is connected to a Fourier domain OCT system based on a swept source laser (AXP50125-6, Axsun Technologies Inc., Billerica, MA, USA), with a central wavelength of 1285 nm, a sweep range of 135 nm and a 100 kHz tuning rate. Thus, 2D OCT cross-sections consisting of 1000 depth scans (A-scans) were detected over a field of view (FOV) of 4 mm with a frame rate (B-scan rate) of 100 fps. Due to the sinusoidal actuation of the fiber optic tip and the fan-shaped scan with an aperture angle of approximately 4.4${}^{\circ }$, the effective FOV is 2.8 mm. The axial resolution of the system is 11 µm and the lateral resolution resulting from the GRIN-based fiber optics is 15 µm (FWHM) [40].

### 2.2. Study Protocol

To evaluate the use of the OCT miniprobe in routine examinations, 15 patients (5 men and 10 women, mean age: 65.8, range: 52–84 years) with visually diagnosed non- and symptomatic oral lichen planus (OLP) and, for differential diagnosis, leukoplakia were included in the observational study. Patient selection took place during the weekly oral mucosal consultation of the Clinic and Polyclinic for Oral and Maxillofacial Surgery of the University Hospital Dresden. In combination with the patient’s symptoms and medical history, the diagnosis of OLP was made by clinical visual observation of the oral mucosal surface of the pathologically altered region and, in two individual cases, supported by biopsy followed by histopathological examination. All patients (≥18 years) with OLP and leukoplakia (including the anterior and posterior buccal mucosa, floor of the mouth, hard palate and tongue) who agreed to undergo non-invasive in vivo OCT imaging of the existing lesion after informed consent were included in the study. Patients with local infectious diseases (e.g., tonsillitis, stomatitis), with multidrug-resistant germs and/or diseases requiring isolation (e.g., tuberculosis) were excluded. Data collection was performed weekly over a two-month period for a total of seven days. Each patient was clinically examined only once during the medical appointment.

Based on the clinical visual diagnosis, 13 patients were diagnosed with oral lichen planus (OLP) (patient I–XIII) and two patients with leukoplakia (patient XIV) and dysplastic leukoplakia (patient XV), respectively. The medical history and medication for each patient are summarized in Appendix A (Table A1, Table A2, Table A3, Table A4, Table A5 and Table A6). For two of the fifteen patients (patient IV and XV), clinical examination required subsequent biopsy and histopathologic examination (based on the hematoxylin and eosin (H&E) staining protocol). For patient IV, the clinical diagnosis of OLP was confirmed histopathologically. In contrast, for the biopsy taken from patient XV, instead of the first preliminary clinical visual diagnosis of dysplastic leukoplakia, the histopathological diagnosis was squamous cell carcinoma.

OCT imaging was performed during the clinical consultation directly after anamnesis and visual–tactile examination and diagnosis by an experienced oral and maxillofacial physician. The decision to take a biopsy was made subsequent to the clinical examination without consideration of OCT imaging. Before OCT cross-sectional imaging, the miniprobe was disinfected (Sekusept Aktiv, Ecolab Deutschland GmbH) and covered with a dental disposable plastic sleeve (X-ray Cover, Henry Schein Inc.) (left part in Figure 1). For each patient, OCT imaging of the physician-determined OLP center and surrounding inconspicuous oral mucosa was obtained by having the miniprobe in light contact with the tissue. In addition, oral alterations were documented by clinical photographs.

### 2.3. Classification Parameter

**Figure 2:**
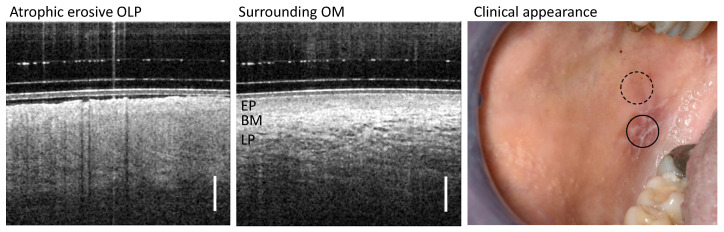
Representative study result of OCT cross-sections of the center of OLP (**left**) and surrounding oral mucosa (OM) (**middle**) of patient XII with clinically diagnosed atrophic erosive OLP. Corresponding clinical appearance (**right**) with marked zones of OCT scanning: OLP center (circle with black solid line) and surrounding OM (circle with black dashed line). Labels: EP: epithelium, BM: basement membrane, LP: lamina propria. Scale bar 500 µm.

Based on clinical appearance, OLP was classified into groups according to the Andreasen classification [41], whereby the types of OLP can occur clinically in isolation or as a mixed subtype. In general, the classification helps to decide on further therapy and diagnosis [42,43]. For our study, the classification of OLP is used to determine class-specific features in OCT cross-sectional images. As a representative example of an OLP mixed subtype, OCT scans of the altered mucosa of a patient with clinically diagnosed atrophic erosive OLP are shown in Figure 2.

In general, OLP is divided into a homogeneous, white form, as well as an inhomogeneous, red form. The homogeneous (hyperkeratinized) form of OLP is often asymptomatic and includes reticular, papular and plaque-like OLP. These asymptomatic OLP forms do not require treatment with glucocorticoids or immunosuppressants, only medical monitoring at regular intervals depending on the variability of the pathology. In addition, supportive measures such as regular professional tooth cleaning, abstaining from nicotine and alcohol, switching to surfactant-free toothpaste and the removal of amalgam fillings are recommended to prevent the worsening of OLP [44,45]. The red forms include erosive (ulcerative), atrophic and bullous forms of OLP, which are commonly symptomatic. Consequently, local immunosuppressive therapy is usually performed, containing glucocorticoid irrigations and adhesive ointments with the aim of the epithelialization and closure of the collapsed epithelial layer [44,45].

With respect to OCT imaging, classification parameters are defined based on previous feasibility studies [29,40]. For unclassified OLP in general, the following structural changes in the oral mucosa compared to the normal state are expected (Table 1), and serve as a basis for the specification of the altered morphology in OCT, dependent on the clinical OLP classification. With this, it becomes possible to derive OLP-specific features in OCT.

## 3. Results

The image data compiled below underlie the classification of the clinical appearance. The aim is to correlate the in vivo depth-resolved cross-sectional OCT image information with the clinical diagnosis.

### 3.1. Reticular OLP

**Figure 3:**
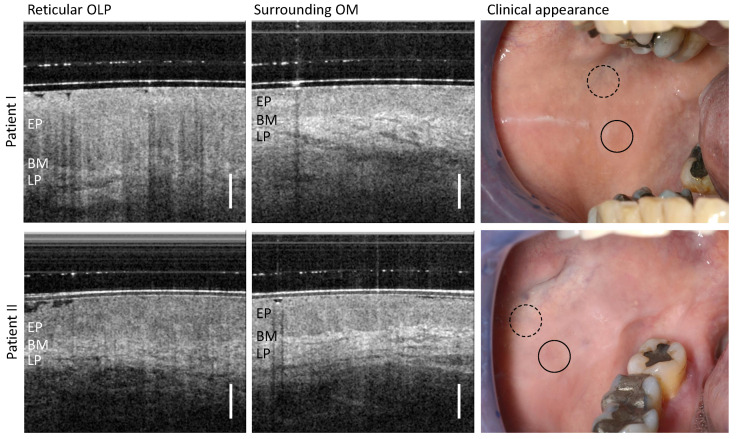
OCT cross-sections of the center of reticular OLP (**left** column) and surrounding oral mucosa (OM) (**middle** column) of patient I (**upper** row) and II (**lower** row). Corresponding clinical appearance (**right** column) with marked zones of OCT scanning: OLP center (circle with black solid line) and surrounding OM (circle with black dashed line). Labels: EP: epithelium, BM: basement membrane, LP: lamina propria. Scale bar 500 µm.

Patient I and II were diagnosed with a reticular form of OLP (Table A1). The visual impression (right column of Figure 3) shows a homogeneous smooth mucosa surface and a whitish reticular keratinization.

The OCT scan detected in the center of the OLP (left column in Figure 3) does not allow a clear demarcation between the epithelium (EP) and the underlying lamina propria (LP), for which reason the course of the basement membrane (BM) can only be partially identified. In addition, the backscattering of the EP appears locally increased and the reflectivity of the collagen fiber network in the LP is reduced. The vascular network within the LP is inconspicuous. These changes become more apparent when compared against an OCT cross-section of the surrounding oral mucosa (OM). There, the EP can be clearly distinguished from the LP and the EP thickness was measured to be 451 µm (patient I) and 528 µm (patient II), using a refractive index of 1.4 for the epithelium at a wavelength of 1300 nm [29,40]. In addition, the vascular structures in the LP are found to be larger and of a lower quantity compared to the OLP center.

### 3.2. Reticular Atrophic OLP

**Figure 4:**
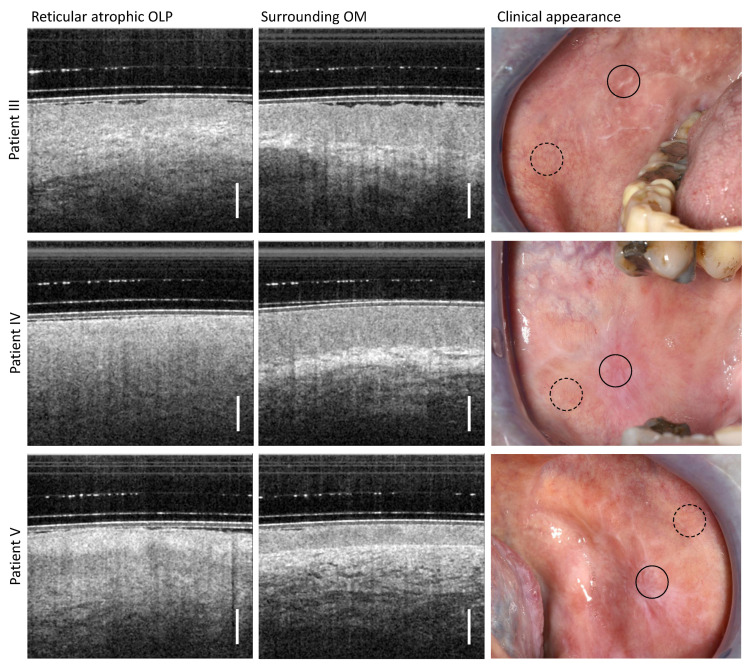
Comparison of OCT cross-sectional images for the center of the reticular atrophic lesion of the oral mucosa (**left** column) and the surrounding region of the buccal mucosa (**middle** column) of patient III (**upper** row), patient IV (**middle** row) and patient V (**lower** row). Photographs for the documentation of the clinical appearance of reticular atrophic OLP with marked locations of OCT imaging: OLP center (circle with black solid line) and surrounding OM (circle with black dashed line). Scale bar 500 µm.

According to the clinical findings, three patients (III, IV, and V) were assigned to the reticular atrophic form of OLP (Table A2), which can be described by an inhomogeneous oral mucosa with whitish distinct reticular keratinization and slightly reddened surface (right column in Figure 4).

For all three patients studied, the OCT imaging depth in the center of OLP is comparable to that of the peripheral region. Due to the structural changes in the distinct OLP, the stratification character of the oral mucosa is only recognizable to a very limited extent in the OCT scans of the lichen center (left column in Figure 4). Thus, for patient III (upper row in Figure 4) and V (lower row in Figure 4), the connective tissue layer with a vascular network can be seen slightly, whereas for patient IV (middle row in Figure 4), the transition is smooth and no layered structure is visible. For patient III, a reduced EP thickness is discernible to a limited extent. For patient V, there is also a pronounced keratinization (reticular hyperkeratiniziation of the EP), which is evident from the increased backscattering of the EP surface. Between this keratinized layer and the underlying connective tissue layer, with embedded small vessels, lies the squamous epithelium with inconspicuous backscattering.

Regarding the surrounding OM, a delineable homogenously scattered EP with a low keratinization of the surface becomes clear. The measured EP thickness of patient III and patient IV is comparable (546 µm for patient III and 574 µm for patient IV), and is reduced for patient V (289 µm). For all three patients, the BM does not form sharply. The vascular network of patient V is slightly increased in the LP with a greater number of smaller vessels.

### 3.3. Atrophic OLP

**Figure 5:**
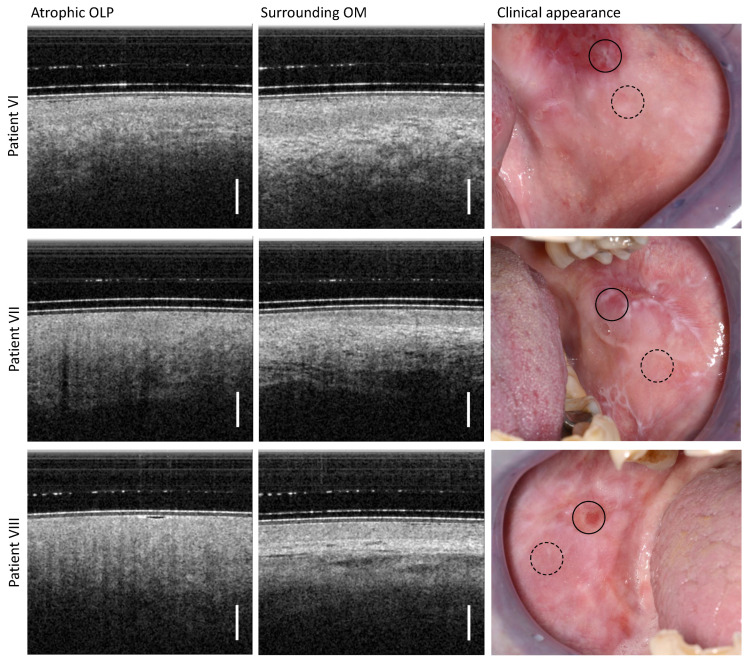
Results of OCT imaging in the center of the atrophic OLP (**left** column) and the surrounding inconspicuous OM (**middle** column) of the patients VI (**upper** row), VII (**middle** row), and VIII (**lower** row). The corresponding photographs of the clinical appearance with OCT labeling are shown in the (**right** column): OLP center (circle with black solid line) and surrounding OM (circle with black dashed line). Scale bar 500 µm.

The findings shown in Figure 5 belong to the atrophic form of OLP with an inhomogeneous and predominantly reddened surface of the mucosa (right column in Figure 5) diagnosed in patient VI, VII and VIII (Table A3).

For OCT imaging, the center of atrophic OLP is characterized by a general hyporeflectivity of OM. There, the differentiation between EP and LP is difficult. The BM as a demarcation line is not visible due to acute inflammation combined with the loss of the EP layer characteristic for this OLP form [41]. In addition, the backscattering of the LP is strongly reduced and an increased number of small vessels become apparent. This correlates with the clinical findings of the atrophic area of the OLP, which appears as a reddened mucosal area of variable size.

The clinically inconspicuous OM surrounding the atrophic OLP shows an expected hyporeflective EP and a hyperreflective LP with embedded larger vessels. The EP has a smaller layer thickness (391 µm for patient VI; 229 µm for patient VII; and 246 µm for patient VIII) than typical for the buccal mucosa area [29]. Both layers, EP and LP, can be reliably distinguished from each other.

### 3.4. Atrophic Erosive OLP

**Figure 6:**
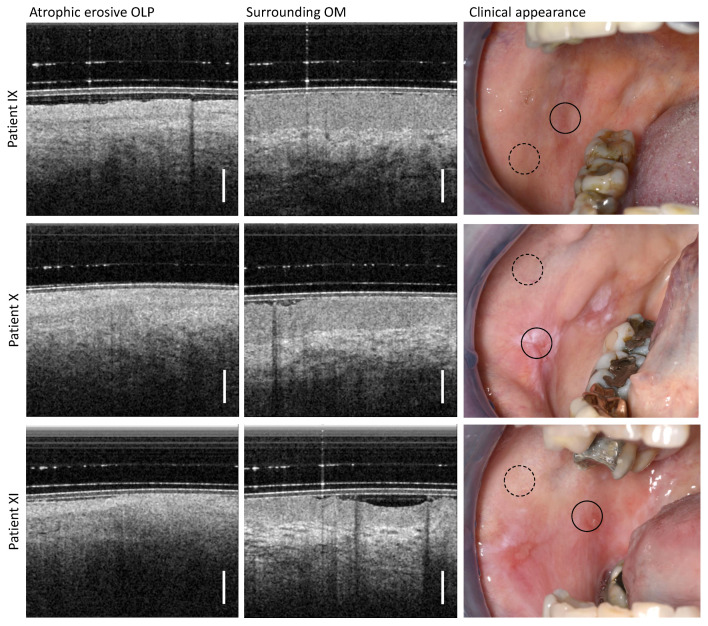
OCT imaging of the center of the atrophic erosive form of OLP (directly adjacent to the open wounds) (**left** column) as well as more distant inconspicuous appearing regions of the buccal mucosa (**middle** column). Three patients (IX, X, XI) were examined, whose clinical findings with marked locations of OCT imaging are shown (**right** column): OLP center (circle with black solid line) and surrounding OM (circle with black dashed line). Scale bar 500 µm.

By clinical palpation with a blunt instrument, the altered oral mucosa of patients IX, X, and XI (Table A4) was found to be atrophic erosive OLP with isolated small (partially fibrin-covered) open ulcerative wounds (right column in Figure 6). This finding is confirmed in the OCT imaging results of the pathological OM adjacent to the open wounds. The EP thickness is reduced and the EP surface is hyperreflective due to distinct keratinization. In addition, the EP is distinguishable from the LP, for which reason the BM is weakly noticeable. The LP is hyporeflective and shows a network of smaller vessels; typical larger vessels are not recognizable.

Peripheral areas at a larger distance from the OLP center show an expected characteristic layering of OM structures with hyporeflective EP and hyperreflective LP with clearly visible larger vessels. The EP thickness amounts to 539 µm (patient IX), 479 µm (patient X) and 363 µm (patient XI) [29,40].

### 3.5. Plaque-like OLP

**Figure 7:**
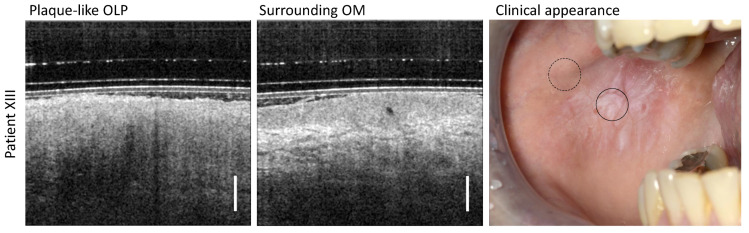
OCT scans of the center of the plaque-like OLP and a measurement site of the surrounding area of patient XIII. Clinical appearance: OLP center (circle with black solid line) and surrounding OM (circle with black dashed line). Scale bar 500 µm.

According to the follow-up of patient XIII (Table A5), the clinical findings were classified as plaque-like homogeneous OLP due to the presence of typical appearance features such as homogeneous whitish keratinization without erosive areas covering large regions of the oral mucosa (Figure 7). The OCT scan in the center of the plaque-like OLP reveals a thin (104 µm) highly hyperreflective layer on the EP surface and an increased reflectivity of the EP itself. In addition, the imaging depth is severely limited, but without shadowing, such that stratification of the OM with a delineable EP and recognizable BM is not possible.

### 3.6. Leukoplakia and OSCC

**Figure 8:**
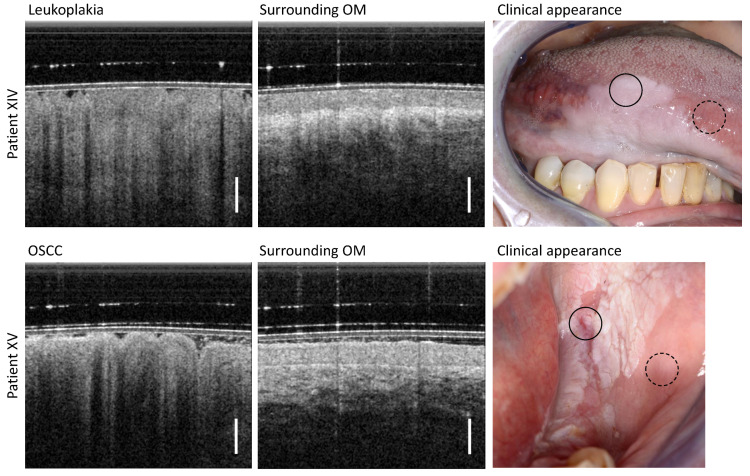
(**Upper** row): Results of patient XIV clinically diagnosed with homogeneous leukoplakia of the ventral tongue. (**Lower** row): Results of patient XV visually diagnosed with homogeneous leukoplakia of the posterior buccal mucosa. The histologic findings of the biopsy taken immediately after OCT imaging revealed that this form of leukoplakia had degenerated into an oral squamous cell carcinoma (OSCC). Scale bar 500 µm. Upper row, leukoplakia (circle with black solid line) and surrounding OM (circle with black dashed line) lower row, OSCC (circle with black solid line) and surrounding OM (circle with black dashed line).

The results of OCT imaging of two patients with visually diagnosed leukoplakia are shown in Figure 8. Patient XIV has homogeneous leukoplakia of the ventral tongue (upper row in Figure 8) and patient XV has inhomogeneous leukoplakia of the posterior buccal oral mucosa (lower row in Figure 8) (Table A6). Beginning with patient XIV, the leukoplakia appears homogeneously closed, covering almost the entire lower surface of the tongue. The corresponding OCT scan of the leukoplakia shows an apparently decreased reflectivity of the entire OM with no decreased OCT imaging depth. Furthermore, the EP surface is uneven and the reflectivity of the EP over depth is inhomogeneous. The underlying adjacent LP can only be delineated uncertainly, as the BM and vascular network of the LP are not clearly identifiable. The OM of the adjacent area of the anterior ventral tongue, not affected by leukoplakia, shows an expected low EP backscattering and an epithelial thickness of 257 µm typical for this oral region [29,40]. In addition, the connective tissue adjacent below the EP is clearly delineated by the high reflectivity of the collagen fiber structures within the LP.

The visually assessed inhomogeneous leukoplakia of the posterior buccal side of patient XV was histologically diagnosed as oral squamous cell carcinoma (OSCC) and appears in the OCT cross-sectional image as particularly striking and different from the previous OCT cross-sectional scans of OLP. In detail, a strongly uneven EP surface and advanced structural remodeling of the EP layer is present. In addition to the highly reduced OCT imaging depth, increased shadowing of the cluster-like circular structures of the EP is also visible. Compared to the OCT depth scans of the different forms of OLP in sections 3.1 to 3.6, the center of this pathology shows neither a decreased stratification of the OM (cp. Figure 3 and Figure 4) nor a homogeneous backscattering behavior with slightly or non-reduced penetration depth (cp. Figure 5, Figure 6 and Figure 7).

The OCT scan of the adjacent visually inconspicuous region (surrounding OM) of patient XV also appears altered: the backscattering of the EP layer is increased and the boundary with the LP appears as a highly backreflective demarcation line above an untypical hyporeflective LP.

### 3.7. Histological Findings

**Figure 9:**
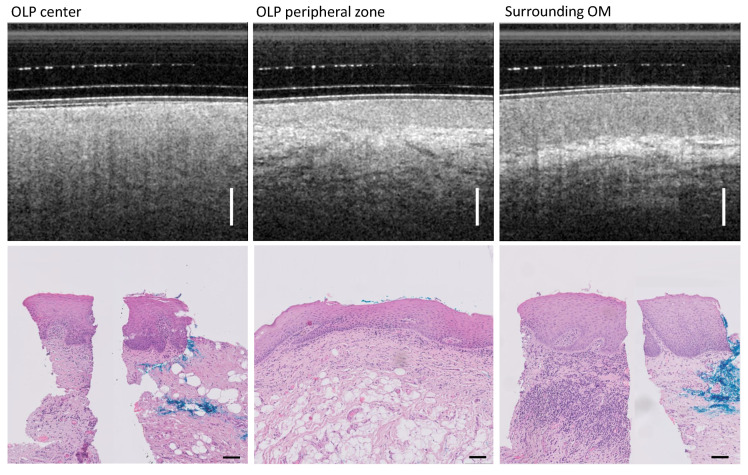
Patient IV with diagnosed reticular atrophic OLP. Comparison of OCT cross-sectional images and H&E-based histopathology results for the three classes: OLP center (**left**), OLP margin (**middle**), and inconspicuous OM in the periphery of the buccal mucosa (**right**). For the OLP center (**lower left**) and surrounding OM (**lower right**), single representative H&E sections have been compounded to one histological overview. The blue color in the tissue sections is used for the spatial orientation of the section during microscopic examination. Scale bar OCT 500 µm, histology 100 µm.

For patients IV and XV, biopsies were taken during consultation, allowing a qualitative comparison to in vivo OCT measurements performed with the miniprobe system. Patient IV was clinically diagnosed with the reticular atrophic form of OLP (Table A2). The three representative OCT scans in Figure 9 show the appearance of the OLP center, OLP margin, and visually normal-appearing surrounding OM. The H&E-stained tissue sections of patient IV were also classified into these three areas and qualitatively compared to the OCT section images. First, a mild epithelial hyperkeratosis is noted for the OLP center, manifested by a narrow hyperreflective area of the epithelial surface on the OCT image. In the corresponding H&E section, a lymphocytic inflammatory infiltrate is evident which is not only distributed in a band-like manner below the epithelial layer, but also penetrates the basement membrane and occasionally reaches the stratum basale as well as the stratum spinosum of the oral squamous epithelium. Additionally, for the OLP center, civatte bodies (apoptotic keratinocytes) are found histologically. For the transitional region between the OLP center and the inconspicuous OM in the periphery, lymphocytes within the lamina propria as well as occasionally infiltrating the stratum basale are evident. There, the lymphocyte infiltrate is less dense. For the surrounding clinically inconspicuous OM, lymphocytes are also present, but these do not infiltrate through the basement membrane into the stratum basale.

**Figure 10:**
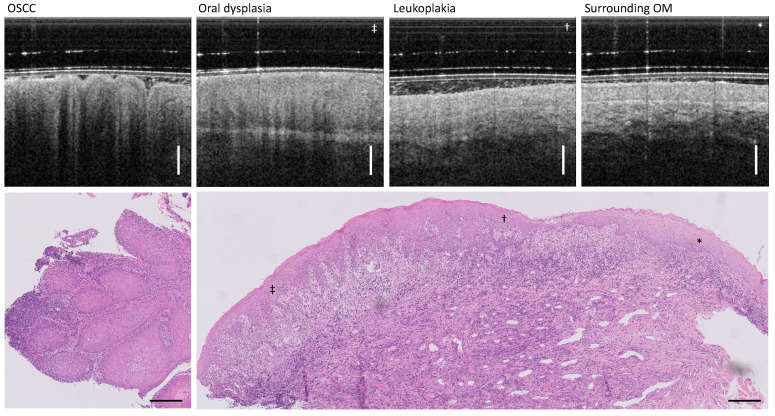
Patient XV with visually diagnosed leukoplakia and histologically found OSCC. Representative OCT cross-sections show the high variance in appearance of altered deeper OM structures, providing helpful information for accurate biopsy sampling in the future. Scale bar OCT 500 µm, histology 250 µm.

For patient XV (Table A6), different areas of visually diagnosed inhomogeneous leukoplakia were imaged. During OCT imaging, the center of the pathologically altered buccal mucosa presented a high variance in appearance; therefore, four representative OCT cross-sections were detected. The pathohistological findings of the biopsy taken in this area revealed an OSCC. Figure 10 lists the recorded OCT scans and compares them to the correlated H&E tissue sections. The large histological section in the lower right clearly shows different areas of clinically diagnosed leukoplakia: (*) shows a distinct lymphocyte infiltrate with intact basement membrane as well as a highly hyperkeratinized epithelial surface. Stratification of the mucosa is evident. (†) shows isolated lymphocytes infiltrated into the stratum basale as well as a distinct band-like subepithelial lymphocyte infiltrate. (‡) also shows civatte bodies (apoptotic keratinocytes) and altered epithelial cells of the stratum basale in addition to the features in (†).

The cloud-like structures of the altered OM in the in vivo OCT cross-section are confirmed in the histological result by prominent circular structures (left column in Figure 10). In the histological section, horn pearls with pleomorphic cells typical for keratinized OSCC are noticeable. In addition to the nuclear pleomorphism of the tumor cells (as a sign of disturbed cell division and enlarged nuclei) and the different degrees of tumor cell maturation, an accumulation of lymphocytes and a ruptured basement membrane are recognizable. The characteristics of OSCC in patient XV are represented by the cloud-like structure of the OM surface in the OCT cross-sectional images. Thus, in the OCT cross-sectional image, there is initially a hyperreflective surface due to hyperkeratinization, which is initially followed in depth by a band of weak signal and below by a band of a stronger backscattering signal. In addition, a pronounced shadowing is evident. The onion-like stratified epithelial pearl in OCT is presumably due to the pathological change in the shape of the epithelial layer and the contained pleomorphic cells as well as partially infiltrated lymphocytes and apoptotic keratinocytes. The alternating shadowing may be attributed to the lymphocyte infiltrate [10]. An exact assignment can only be proven by further studies.

## 4. Discussion

In the presented study, patients with visually diagnosed clinical non- and symptomatic OLP and, for differential diagnosis, leukoplakia were imaged with OCT in vivo during the routine annual follow-up. The clinically classified forms and mixed forms of OLP were correlated with the depth-resolved in vivo insight into the altered OM. The morphological information provided by OCT augments the clinical appearance and may provide the opportunity for more accurate localization of a representative biopsy, if needed. Using the small sample of patients in our study, we extended the clinical diagnosis by the corresponding characteristic features of OCT imaging (see Table 2). These include EP integrity, EP thickness [29,46], the identifiability of the BM and the vascular network within the connective tissue [29], as well as the reflectivity of the EP and the LP, and the associated differentiability of the near-surface mucosal layers. Comparable morphographic patterns with fewer features have recently been proposed for erosive and bullous OLP in a study of OLP-related immune-mediated gingivitis [47]. By means of the results presented here, it becomes apparent that with the visualization of the intact or degenerated layered structure of the oral mucosa, the type as well as the progression of OLP can be specified more accurately in combination with the clinical appearance.

These results were confirmed by previous studies [38,39,40,47]. Gambino et al. previously described the OCT imaging of the atrophic-erosive form of OLP and investigated the structural changes in the BM and vascular network by means of a commercial swept source OCT dermatological instrument complemented by a customized probe designed for oral soft tissue imaging. The features observed in [38,39] are a disruption of the BM and irregular vascularization, reinforced by the findings of our study which additionally considered other morphological variants of OLP. A comparable commercial OCT system was used in a study of immune-mediated gingivitis in association with erosive and bullous OLP [47] where OCT sectional images were matched to histologic sections, with the result of a reduced EP and a BM alteration due to inflammatory infiltrate. Consequently, the merit of the present study is the overview of the appearance of degenerated oral mucosa of different forms of OLP on intensity-based OCT and the derived definition of the enhanced OCT-specific degeneration features.

From the clinical perspective, the results of this study, as well as the findings of previous publications, suggest that OCT might complement the visual examination of the oral mucosa at the initial clinical consultation and at the annual follow-up. The example of patient XV, initially clinically diagnosed as inhomogeneous dysplastic leukoplakia but later found to be an OSCC on biopsy, illustrates the possible added value of OCT for valid biopsy sampling. In this context, OCT imaging could potentially serve as an additional decision aid to determine whether a control biopsy should be performed or whether the patient should be readmitted. Concerning, for example, oral leukoplakia, OCT imaging might also support the differentiation of OLP from other oral lesions [17]. Furthermore, according to previous studies [34,35,36], OCT may help in detecting dysplastic epithelial areas within extensive lesions in order to guide more precise biopsies. However, it must be recognized that the identification and assessment of oral dysplasia can, at the moment, only be achieved with a lower sensitivity and especially specificity compared to invasive oral cancer [35,36].

With regard to future research, it will be necessary to extend the OCT imaging of OLP to a larger number of patients to validate the results of the present study. Thereby, the variants of the erosive OLP and especially the more treatment-intensive red forms of OLP should be focused on. In addition to in vivo OCT imaging, the ex vivo imaging of biopsy specimens in combination with consecutive histopathological evaluation may also be useful to further correlate OCT and histological findings more accurately [29,40,47]. Moreover, it is conceivable to expand the so far qualitative assessment of mucosal changes induced by OLP by a set of quantitative criteria in order to enable a more objective and preferably automated analysis of the OCT images in the future. The angiographic analysis of OCT volume data as well as depth-resolved birefringence to contrast subepithelial connective tissue might be valuable [48,49]. Finally, with respect to the current state of knowledge, it can be ascertained that OCT has the potential to improve routine examinations of oral mucosal lesions and to increase the precision of biopsy collection for reliable diagnosis.

## 5. Summary and Conclusions

Any form of OLP can degenerate malignantly over time. In various well-conducted studies, data on the malignant transformation rate of OLP range from 0.3% to 14.3% with an average of 1.1% and a median follow-up of about 5.5 years [9,11,15]. Malignant transformation can occur in all clinical types and mixed forms of OLP [12,14]. Transformation rates vary among different types of OLP, with erosive and atrophic types (1.7% and 1.3%, respectively) having higher rates compared to the reticular type (0.1%) [15]. The reliable diagnosis of OLP in clinical practice is ensured by visual tactile examination by an experienced physician followed by histologic evaluation as the gold standard for assessing the malignant transformation risk of OLP based on morphological changes. Re-sampling of a biopsy in the follow-up of OLP patients is necessary whenever the clinical presentation changes and becomes suspicious. However, the transition from non-suspicious to suspicious is sometimes difficult to define clinically, as is the correct site for biopsy in an extensive widespread oral lesion. Non-invasive optical biopsy may help in this regard in the future. As a first step in the verification of this hypothesis, in the present case-series study, OCT cross-sectional images of the center and marginal areas of different types and subtypes of OLP were obtained non-invasively and correlated with the visual clinical findings and, if available, with histopathology. By identifying and describing OLP-specific features in OCT cross-sections for various forms of OLP, we provide a basis for the non-invasive differentiation of suspicious dysplastic lesions from OLP as a future aid for improved biopsy sampling and to ensure reliable histopathologic assessment. The next step is to further investigate the proposed OCT technology in clinical studies with a larger number of cases and to establish valid data for the sensitivity and specificity of OCT for the differentiation of various OLP forms and in distinction from dysplastic changes, as well as OSCC.

## Figures and Tables

**Table 1:** Definition of parameters for the evaluation of OCT sectional images dependent on the clinical OLP classification.

Parameter	Normal OM	Pathological OM
EP thickness ^1^	distinct thickness	reduced thickness
Basement membrane	distinct demarcation	less/none demarcation
EP reflectivity ^2^	highly translucent	altered backscattering
LP reflectivity ^3^	highly reflective	reduced backreflection
Vascular network in LP	identifiable vascularization	altered vascularization

^1^ Epithelial (EP) thickness. ^2^ Epithelial (EP) reflectivity. ^3^ Reflectivity of the Lamina propria (LP).

**Table 2:** Summary of OCT features compared with clinical appearance for different forms of OLP in comparison to leukoplakia and OSCC by means of a clinical study of N = 15 patients.

Pathology	Clinical Appearance	OCT Features
Reticular OLP		increased EP surface reflectivity
	homogeneous smooth surface	reduced LP reflectivity
	whitish keratinization	limited BM visibility
		inconspicious vessel network
Reticular atrophic OLP		EP surface hyperreflectivity
	inhomogeneous OM surface	reduced EP thickness
	whitish reticular keratinization	reduced LP reflectivity
	marginal reddened surface	vague BM visibility
		reduced layer differentiation
		reduced nb. of large vessels
Atrophic OLP		EP hyporeflectivity
	inhomogeneous OM surface	no BM visibility
	predominant reddened surface	no layer differentiation
		enhanced vessel network
Atrophic erosive OLP		EP hyperreflectivity
	hyperkeratinization	reduced EP thickness
	ulcerative open wounds	limited BM visibility
	fibrin layer	reduced layer differentiation
		reduced nb. of large vessels
Plaque-like OLP		hyperreflective layer at EP
	hyperkeratinization	no BM visibility
	large whitish homogenous OM	no layer differentiation
		no vessel network identification
Leukoplakia		hyporeflective surface
	homogenous/inhomogeneous	distinct shadowing
	closed hyperkeratinization/	no BM visibility
	interrupted hyperkeratinization	no layer differentiation
		no vessel network identification
OSCC		hyperreflective EP surface
	uneven interrupted surface	strongly reduced imaging depth
	floe-like hyperkeratinization	cloud-like layered structure
	distinct ulceration	distinct shadowing
		no BM and layer visibility
		no vessel network identification

## Data Availability

The data presented in this study are available on request from the corresponding author.

## Abbreviations

The following abbreviations are used in this manuscript:

A-scan	Amplitude scan (1D OCT depth scan)
B-scan	Brightness scan (2D OCT scan, cross-section)
GRIN	Gradient index
FOV	Field of view
FWHM	Full width half maximum
OC	Oral cancer
OCT	Optical coherence tomography
OSCC	Oral squamous cell carcinoma
OLP	Oral lichen planus
OM	Oral mucosa
EP	Epithelium
LP	Lamina propria
BM	Basement membrane

## Appendix A

**Table A1:** Anamnesis of patients with clinically found reticular OLP.

Nb	Gender	Age	Disorders	Medication
I	female	67	OLP: left buccal mucosa mouth xerostomia (dry mouth); thrombosis; thyroid and stomach disorder; keratoconjunctivitis sicca (eye dryness); osteoporosis; removed ovaries	Hydrocortisone oral ointment
II	female	60	OLP: right buccal mucosa; hypertension; thyroid disorder;	Losartan-ratiopharm 50 mg (antihypertensive drug); Triamcinolone ointment 0.1% (glucocorticoid); Micotar ointment (antifungal drug)

**Table A2:** Anamnesis of patients with clinically found reticular atrophic OLP.

Nb	Gender	Age	Disorders	Medication
III	male	77	OLP: right buccal mucosa; hypertension; unusual bleeding or bruising; osteoporosis; asthma; COPD; birch allergy; immunodeficiency because of the prostate cancer irradiation (2011); gallbladder operation	Antihypertensive drugs; Pantoprazol
IV	male	80	OLP: right buccal mucosa; hypertension	Antihypertensive drugs; Celestone Liquidum 0.5 mg/mL N1 (glucocorticoid drug)
V	female	52	OLP: left buccal mucosa hypertension; allergy to benzoyl peroxide, adhesives, ceramic restorations, perfume fragrances	antihypertensive drug-VIACORAM 7 mg/5 mg; Celestone Liquidum 0.5 mg/mL N1 (glucocorticoid drug)

**Table A3:** Anamnesis of patients with clinically found atrophic OLP.

Nb	Gender	Age	Disorders	Medication
VI	male	64	OLP: left buccal mucosa; hypertension; thyroid disorder; removal of the thyroid (1990); allergy (hay fever)	L-Thyroxin; Amlodipin; Enalapril; Simvastatin; Omeprazol; Triamcinolone injections
VII	male	60	OLP: right buccal mucosa	Triamcinolon acetat 0.1; Micotar ointment
VIII	female	82	OLP: right buccal mucosa; Hypertension; Circulatory disorder; Diabetes mellitus type 2; Osteoporosis; Rheumatoid arthritis; Allergy to analgesics and metals	Celestone Liquidum 0.5; Micotar ointment; Celebrex; Carmen; Carvedilol; Candecor; Forasamid; Dekristol; Venostatin

**Table A4:** Anamnesis of patients with clinically found atrophic erosive OLP.

Nb	Gender	Age	Disorders	Medication
IX	female	69	OLP: right buccal mucosa; Hypertension; Heart pacemaker; Secondary haemorrhage; Diabetes mellitus type 2; Asthma; Thyroid disorder; Myositis; Hephritis; Allergy to AB, Latex, food; Rheumatoid arthritis	Triamcinolon acetat 0.1%; Micotar ointment; Analgesics; Antihypertensive drugs; Antirheumatic agent
X	female	84	OLP: right buccal mucosa (erosive); unusual bleeding or bruising; artificial joints since 2009 and 2013 (hip joint); glaucoma; intestine; gallbladder operation; asthma; chronic bronchitis; allergy to latex and opiates	Cortisone spray
XI	female	51	n/a	n/a
XII	female	67	OLP: right buccal mucosa	Triamcinolone ointment 0.1% (glucocorticoid); Micotar ointment (antifungal drug)

**Table A5:** Anamnesis of the patient with clinically found plaque-like OLP.

Nb	Gender	Age	Disorders	Medication
XIII	female	48	OLP: right buccal mucosa; operation of the right arm	Propolis; clove oil; Ceterizin (anti-histamine agent)

**Table A6:** Anamnesis of patients with clinically found leukoplakia.

Nb	Gender	Age	Disorders	Medication
XIV	female	60	Leukoplakia: tongue (right side); hypertension; varicose veins operation (left side)	Valsacor
XV	male	66	Leukoplakia: left buccal mucosa and regio 37–38; hypertension; heart attack; apoplexy; thrombosis; unusual bleeding or bruising; secondary bleeding; diabetes mellitus type 2; thyroid disorder; artificial joints since 2014	Insulin; Antihypertensive drug
